# Posterior cruciate ligament injuries: what do we really know?

**DOI:** 10.1007/s00167-020-06425-3

**Published:** 2021-01-23

**Authors:** Philipp W. Winkler, Jonathan D. Hughes, James J. Irrgang, Jón Karlsson, Volker Musahl

**Affiliations:** 1 Department of Orthopaedic Surgery, UPMC Freddie Fu Sports Medicine Center, University of Pittsburgh, 3200 S. Water St, Pittsburgh, PA 15203 USA; 2 Department for Orthopaedic Sports Medicine, Klinikum Rechts der Isar, Technical University of Munich, Ismaninger Str. 22, 81675 Munich, Germany; 3 Department of Physical Therapy, University of Pittsburgh School of Health and Rehabilitation Sciences, Suite 210, 100 Technology Drive, Pittsburgh, PA 15219 USA; 4 Department for Orthopaedics, Sahlgrenska University Hospital, Institute of Clinical Sciences, Sahlgrenska Academy, Gothenburg University, Gothenburg, Sweden

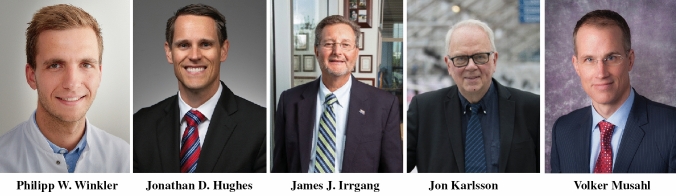



Severe anterior knee pain, an increasing feeling of instability, and a striking hyperextension-varus thrust [16] (i.e., triple varus) clearly demonstrated the devastating effects of recurrent posterior cruciate ligament (PCL) and posterolateral corner (PLC) insufficiency in a recently treated patient in the outpatient clinic of the Department of Orthopedic Surgery in Pittsburgh. In an effort to provide the best and most evidence-based scientific knowledge, we felt compelled to review the literature and present the current standard of care for isolated, combined, and recurrent PCL injuries [21, 22]. The comprehensive literature review has opened our eyes. What do we actually know about PCL graft failures and recurrent instability? Was the cause of surgical PCL graft failure in the presented patient an inappropriate graft choice, non-anatomic tunnel placement, underlying bony deformities, wrong timing, non-compliance of the patient, bad luck, or even the decision for operative treatment?

Let’s go back to the basics. The central pivot of the human knee joint is represented by the cruciate ligaments, providing not only translational but also rotatory knee stability [8]. While research on the anterior cruciate ligament (ACL) is constantly increasing over the past 40 years, research until the early 1990s hardly focused on the ACL’s big brother—the PCL—which is thicker, stronger, and represents the primary restraint against posterior tibial translation [1, 11, 12]. Most probably, we can learn something from the intensive research and the already established knowledge about the ACL to translate this into the management of PCL injuries.

What has Freddie Fu taught us about the ACL? “*Respect nature”*. Consequently, years of hard work and intensive research have enhanced our knowledge. Isometric ACL reconstruction has been replaced by anatomic ACL reconstruction, and more recently, individualized anatomic ACL reconstruction combines all our knowledge of functional anatomy, grafts and individual bony morphology to good clinical outcomes [9, 15]. *“Respect the past and embrace the future”*. How can we use this advice and translate it to the management of PCL injuries is an important question?

While the concept of anatomic double-bundle ACL reconstruction has not been proven to be superior to individualized anatomic single-bundle ACL reconstruction [10], this may be different for PCL reconstruction. There is biomechanical evidence that has demonstrated favorable results in restoring native knee kinematics and laxity for double-bundle vs. single-bundle PCL reconstruction [6]. Double-bundle PCL reconstruction requires thorough anatomical knowledge for accurate tunnel placement, and although biomechanical evidence exists, we are still waiting for clinical proof that double-bundle PCL reconstruction is superior to single-bundle PCL reconstruction [13, 23]. Accordingly, the concept of graft configuration (single bundle vs. double bundle) may be of secondary importance, while the graft choice (autograft vs. allograft) may be of primary importance. At least in ACL reconstruction, it can be observed that autografts and especially quadriceps tendon autografts are becoming increasingly popular due to low surgical graft failure rates [5, 19].

When we respect the nature, it is also crucial to take a close look at the individual bony morphology. Biomechanical evidence supports that frontal and sagittal lower limb malalignment affect the stress experienced by the PCL and PLC grafts [3]. It has been shown that more than 30% of PCL reconstruction failures are associated with varus malalignment [17]. Concurrent realignment osteotomies may improve functional outcomes and increase arthroplasty-free survival, by protecting concurrent ligament reconstructions and preventing subsequent meniscal and cartilage injuries [3, 18, 20]. Therefore, we call for the assessment and, if necessary, correction of frontal and sagittal lower limb deformities in the operative management of PCL injuries.

There is one more topic to discuss. Should we even recommend PCL reconstruction or is a non-operative treatment including functional bracing, intensive physical therapy, and neuromuscular retraining sufficient? In patients with symptomatic grade three PCL injuries, YES, we recommend PCL reconstruction. Especially in young and active patients to avoid subsequent meniscal and cartilage injuries and a rapid development of osteoarthritis [2]. The far more controversial question, however, is: what is the ideal timing for PCL reconstruction (early vs. delayed)? While evidence supports early reconstruction in patients with ACL injuries, research continues to find answers to this controversial question in patients with PCL and multiple ligament knee injuries [4, 7, 14]. One such research project is the STaR Trial (Surgical Timing and Rehabilitation of multiple ligament knee injuries). The STaR Trial is a large-scale multicenter randomized controlled trial that aims at assessing the effect of timing of surgery, early vs. delayed, and timing of rehabilitation, again early vs. delayed on clinical outcome and return to military and civilian duty/sport. The STaR Trial is being conducted across 25 centers in North America with the University of Pittsburgh as the principal site [14].

This editorial highlights numerous controversies in the management of PCL injuries and appeals to all basic scientists and clinical researchers to continue their vigorous efforts to find more evidence. Given the low incidence of PCL injuries, large-scale studies with a high level of evidence (i.e., randomized controlled trials) are currently missing/on the way. Large-scale prospective clinical studies are required to shed light on controversial topics. Never forget, *“Respect the past and embrace the future”.* Therefore, we look with great curiosity into the future of PCL research.

## Abbreviations

ACL: Anterior cruciate ligament
PCL: Posterior cruciate ligament
PLC: Posterolateral corner
